# A cinnamaldehyde Schiff base of *S*-(4-methyl­benz­yl) di­thio­carbazate: crystal structure, Hirshfeld surface analysis and computational study

**DOI:** 10.1107/S2056989017003991

**Published:** 2017-03-21

**Authors:** Enis Nadia Md Yusof, Mohamed I. M. Tahir, Thahira B. S. A. Ravoof, Sang Loon Tan, Edward R. T. Tiekink

**Affiliations:** a Department of Chemistry, Faculty of Science, Universiti Putra Malaysia, 43400, UPM Serdang, Selangor Darul Ehsan, Malaysia; b Research Centre for Crystalline Materials, School of Science and Technology, Sunway University, 47500 Bandar Sunway, Selangor Darul Ehsan, Malaysia

**Keywords:** crystal structure, hydrogen bonding, di­thio­carbazate ester, Hirshfeld surface analysis, DFT

## Abstract

The title di­thio­carbazate ester (I), C_18_H_18_N_2_S_2_ [systematic name: (*E*)-4-methyl­benzyl 2-[(*E*)-3-phenyl­allyl­idene]hydrazinecarbodi­thio­ate, comprises an almost planar central CN_2_S_2_ residue [r.m.s. deviation = 0.0131 Å]. The methyl­ene(tolyl-4) group forms a dihedral angle of 72.25 (4)° with the best plane through the remaining non-hydrogen atoms [r.m.s. deviation = 0.0586 Å] so the mol­ecule approximates mirror symmetry with the 4-tolyl group bis­ected by the plane. The configuration about both double bonds in the N—N=C—C=C chain is *E*; the chain has an all *trans* conformation. In the crystal, eight-membered centrosymmetric thio­amide synthons, {⋯HNCS}_2_, are formed *via* N—H⋯S(thione) hydrogen bonds. Connections between the dimers *via* C—H⋯π inter­actions lead to a three-dimensional architecture. A Hirshfeld surface analysis shows that (I) possesses an inter­action profile similar to that of a closely related analogue with an *S*-bound benzyl substituent, (II). Computational chemistry indicates the dimeric species of (II) connected *via* N—H⋯S hydrogen bonds is about 0.94 kcal mol^−1^ more stable than that in (I).

## Abstract 2

The title di­thio­carbazate ester has approximate mirror symmetry with the putative plane bis­ecting the –CH_2_(tolyl-4) residue. The configuration about each double bond in the N—N=C—C=C chain is *E*; the chain has an all *trans* conformation. In the crystal, N—H⋯S and C—H⋯π inter­actions link the mol­ecules into a three-dimensional network.

## Chemical context   

A large number of studies have been carried out since 1974 on di­thio­carbazate-derived Schiff bases of general formula NH_2_NHC(=S)S*R* which are synthesized from the condensation reaction of *S*-alkyl or -aryl esters of di­thio­carbazic acid with different types of aldehydes or ketones (Ali & Livingstone, 1974 ▸; Ravoof *et al.*, 2010 ▸; Hamid *et al.*, 2016 ▸). Recent work has reported electrochemical studies of conjugated copper(II) di­thio­carbazate complexes that undergo an irreversible oxidation/reduction of Cu^II^/Cu^I^ (Blower *et al.*, 2003 ▸; Paterson *et al.*, 2010 ▸). Di­thio­carbazate Schiff bases have also been reported to show variable cytotoxicity against estrogen receptor positive human breast cancer cells (MDA-MB-231) and other cell lines depending on their substituents (Pavan *et al.*, 2010 ▸; Low *et al.*, 2016 ▸). In fact, related 2-acetyl­pyridine Schiff bases of *S*-methyl- and *S*-benzyl-di­thio­carbazate have better cytotoxic potential as compared to their complexes (Hamid *et al.*, 2016 ▸). As part of an on-going study on the potential biological activities and structural chemistry of di­thio­carbazate Schiff bases and their metal complexes (Yusof, Ravoof, Jamsari *et al.*, 2015 ▸; Yusof, Ravoof, Tiekink *et al.*, 2015 ▸; Low *et al.*, 2016 ▸), the synthesis of the title compound, (I), its crystal and mol­ecular structures along with an analysis of its Hirshfeld surface and computational modelling are reported herein.
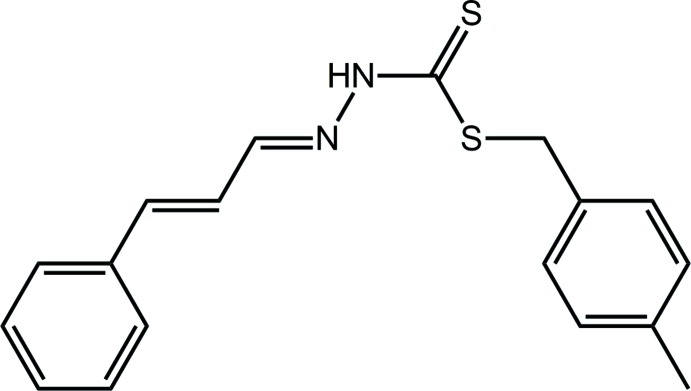


## Structural commentary   

**Figure 1:**
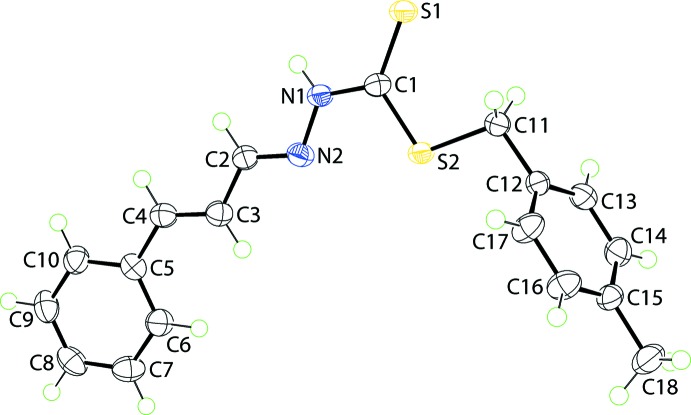
The mol­ecular structure of (I) showing the atom-labelling scheme and displacement ellipsoids at the 70% probability level.

The mol­ecular structure of (I), Fig. 1 ▸, comprises three distinct residues with the central CN_2_S_2_ group being essentially planar with an r.m.s. deviation of the fitted atoms being 0.0131 Å. Appended to this at the S2 atom is a CH_2_(tolyl-4) residue [r.m.s. deviation = 0.0192 Å], and at N2, *via* a C2=N2 imine bond, is a C(H)—C(H)=C(H)Ph group [r.m.s. deviation = 0.0191 Å]. The dihedral angles between the central group and the S2- and N2-bound substituents are 71.65 (4) and 7.08 (8)°, respectively. The dihedral angle between the outer groups is 72.33 (4)° and is indicative of an approximately orthogonal relationship. Indeed, the r.m.s. deviation of all non-hydrogen atom in (I) except those comprising the CH_2_(tolyl-4) residue is 0.0586 Å, and the angle between this plane and that through the CH_2_(tolyl-4) residue is 72.25 (4)°. The 1,4-carbon atoms of the 4-tolyl ring lie on the approximate mirror plane defined by the rest of the mol­ecule with the remaining pairs of ring atoms being related across the putative plane.

The configuration about the C2=N2 imine [1.284 (2) Å] and C3=C4 ethene [1.339 (2) Å] bonds is *E* in each case. This implies the N1—N2=C2—C3=C4 sequence has an all *trans* conformation as seen in the N1—N2—C2—C3, N2—C2—C3—C4 and C2—C3—C4—C5 torsion angles of 177.41 (13), −178.70 (15) and 178.23 (15)°, respectively. The C1—S2 [1.7455 (16) Å] and, especially, C11—S2 [1.8233 (16) Å] bond lengths are considerably longer than the C1—S1 bond [1.6752 (16) Å] consistent with considerable thione character in the latter. This is borne out also by the observation that the angles about the C1 atom involving S1 are wider, by over 7°, *i.e*. S1—C1—S2 = 125.20 (10)° and N1—C1—S1 121.06 (12)°, *cf*. N1—C1—S2 of 113.74 (11)°.

Further discussion on the mol­ecular geometry of (I) is given in *Computational chemistry calculations*.

## Supra­molecular features   

**Figure 2:**
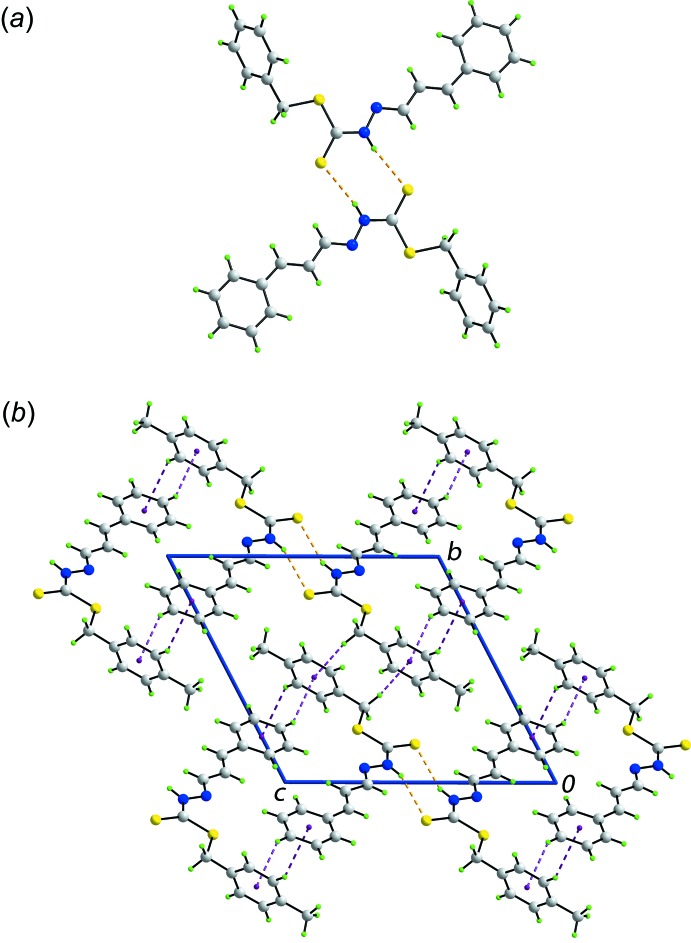
Mol­ecular packing in (I): (*a*) a view of the supra­molecular dimer sustained by N—H⋯S(thione) hydrogen bonds and (*b*) a view of the unit-cell contents shown in projection down the *a* axis. The N—H⋯S and C—H⋯π inter­actions are shown as orange and purple dashed lines, respectively.

The most prominent feature of the mol­ecular packing is the formation of an eight-membered, centrosymmetric thio­amide synthon, {⋯HNCS}_2_ mediated by N—H⋯S(thione) hydrogen bonds, Fig. 2 ▸ *a* and Table 1 ▸. The dimeric aggregates thus formed are connected into a three-dimensional architecture, Fig. 2 ▸ *b*, *via* methyl­ene-C—H⋯π(tol­yl), tolyl-C—H⋯π(phen­yl) and phenyl-C—H⋯π(tol­yl) inter­actions, Table 1 ▸, indicating the tolyl ring accepts two such contacts. In essence, the C—H⋯π inter­actions connect mol­ecules into layers in the *bc* plane and these are linked by the N—H⋯S hydrogen bonds.

## Analysis of the Hirshfeld surfaces   

The most closely related compound in the crystallographic literature is one with a benzyl substituent at the S2 atom (Tarafder *et al.*, 2008 ▸) rather than a CH_2_(tolyl-4) group, that might be regarded as the ‘parent’ compound, hereafter referred to as (II). While detailed discussion on the comparison of their mol­ecular geometries and computational modelling are given in *Computational chemistry calculations*, the present section focuses upon the study of inter­molecular inter­actions formed by (I) and (II) in their respective crystals by Hirshfeld surface analysis in accord with the method described recently (Yeo *et al.*, 2016 ▸).

**Figure 3:**
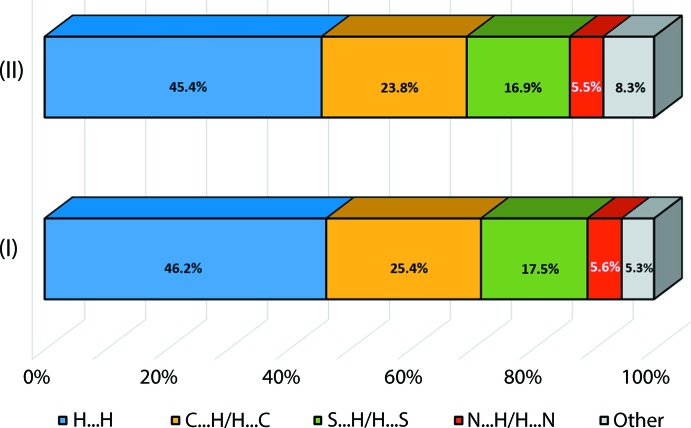
Relative percentage contributions of close contacts to the Hirshfeld surfaces of (I) and (II).

Both (I) and (II) exhibit closely related topological inter­actions as evidenced by the relative distribution of similar contacts, Fig. 3 ▸, computed based upon the mapping of the contact distances at specific points on their Hirshfeld surfaces (Spackman & Jayatilaka, 2009 ▸). Among the inter­actions, H⋯H contacts constitute the most dominant contacts in (I) and (II) at approximately 46.2 and 45.4%, respectively. This is followed by C⋯H/H⋯C [*ca* 25.4% for (I) and 23.8% for (II)], S⋯H/H⋯S [*ca* 17.5 and 16.9%], N⋯H/H⋯N [*ca* 5.6 and 5.5%] as well as other minor inter­actions including N⋯C/C⋯N, S⋯C/C⋯S and S⋯N/N⋯S, which constitute less than 5% of the overall contacts.

**Figure 4:**
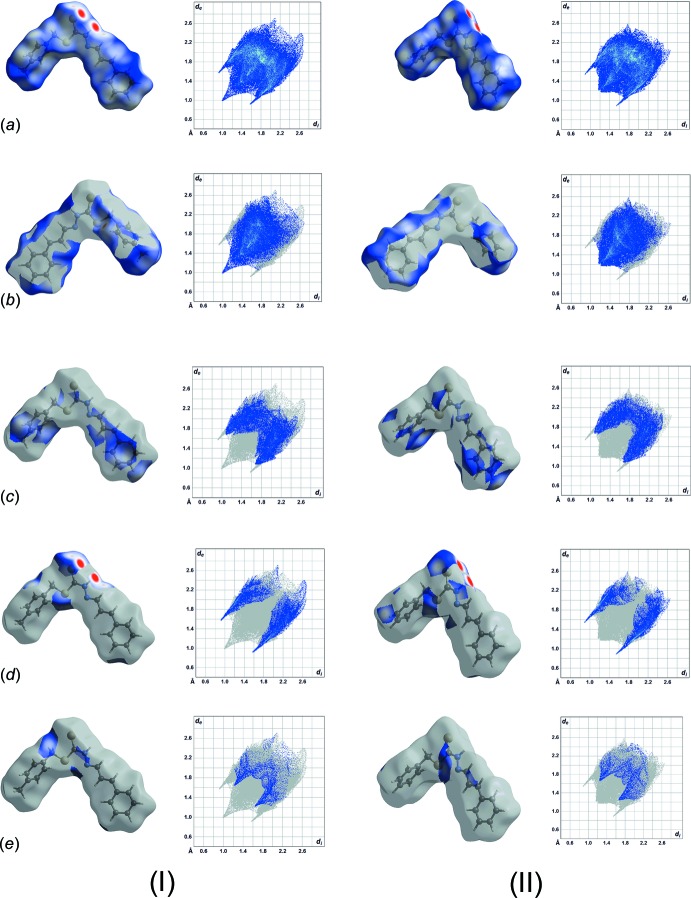
Fingerprint plots for (I) and (II): (*a*) overall and those delineated into (*b*) H⋯H, (*c*) C⋯H/H⋯C, (*d*) S⋯H/H⋯S and (*e*) N⋯H/H⋯N contacts. Note that the Hirshfeld surface showing H⋯H contacts for (I) and (II) are illustrated in the reverse orientation so as to show the close contacts.

A detailed comparison of the two-dimensional fingerprint plots of *d*_i_ *vs d*_e_ at the inter­vals of 0.01 Å reveals that (I) and (II) are qu­anti­tatively different, despite both having a wasp-shape full fingerprint and similar Hirshfeld surface profiles, Fig. 4 ▸ *a*,. Specifically, the decomposed fingerprint plot of H⋯H for (I) displays a *d*_e_ + *d*_i_ contact distance of 1.96 Å which is approximately 0.43 Å (17%) shorter *cf*. 2.36 Å for (II), Fig. 4 ▸ *b*. Both (I) and (II) possess similar C⋯H/H⋯C contact distance, Fig. 4 ▸ *c*, at approximately 2.7 Å, which is slightly shorter than the van der Waals radii of 2.9 Å. The decomposed fingerprint plots of S⋯H/H⋯S (Fig. 4 ▸ *d*) and N⋯H/H⋯N contacts (Fig. 4 ▸ *e*) for (I) register contact distances of 2.47 and 2.90 Å, respectively, which is about 0.05 Å (1.7–2.0%) longer than those of (II). It is noteworthy that the H⋯H contact of (I) is significantly shorter than the sum of their van der Waals radii, by 0.44 Å (22.4%) *cf*. (II), in which the difference is merely 0.04 Å (1.7%). Similarly, the S⋯H/ H⋯S contacts of both (I) and (II) exhibit shorter contact distances *cf*. the sum of their van der Waals radii by 0.53 and 0.58 Å, respectively (21.5 and 24.0%). As a result, those contacts display intense red spots on their Hirshfeld surface, Fig. 4 ▸ *d*.

In view of the close structural similarity between (I) and (II), their physical properties such as mol­ecular volume, surface area, shape, density and packing efficiency were computed either by *Crystal Explorer* (Wolff *et al.*, 2012 ▸) or *PLATON* (Spek, 2009 ▸) and data are compared in Table 2 ▸. As expected, the mol­ecule of (I), which has an additional methyl group *cf*. (II), exhibits a greater mol­ecular volume and surface area, and is slightly less globular. This results in a lower surface-to-volume ratio and density for (I), and ultimately leads to reduced packing efficiency when compared to (II).

## Database survey   

As mentioned in the previous section, the ‘parent’ compound represents the most closely related analogue to (I) in the Cambridge Crystallographic Database (Groom *et al.*, 2016 ▸) and hence, it is adopted for direct comparison in terms of their geometric parameters; selected data are collated in Table 3 ▸. All bond lengths are equal within experimental error and bond angles agree to within 1°. The influence, if any, upon the mol­ecular conformation exerted by the tolyl substituent in (I) might be manifested in the twists about the C11—C12 bond as the S2—C11—C12—C13 torsion angles vary between 3–6°. Equivalent twists are also noted about the C5—C6 bond.

## Computational chemistry calculations   

Both (I) and (II) were subjected to geometry optimization calculations assuming a gas-phase environment in order to compare the structural difference between the experimental and theoretical models. The corresponding theoretical models were first drawn using *GaussView5* (Dennington *et al.*, 2009 ▸) based on the geometrical conformation of the structure (*trans–cis* along C1=S1 and *E*, *E* along N2—C2, C3—C4) and pre-optimized using a semi empirical method (PM6) with a precise self-consistent field criterion. Subsequently, the geometries were further optimized at B3LYP/6-311+G(*d,p*) without imposing symmetry constraints. A frequency analysis was performed on each optimized structure using the same level of theory and basis set to validate that each structure was indeed the local minimum structure with no imaginary frequency. All calculations were performed using the *Gaussian09* software package (Frisch *et al.*, 2016 ▸).

**Figure 5:**
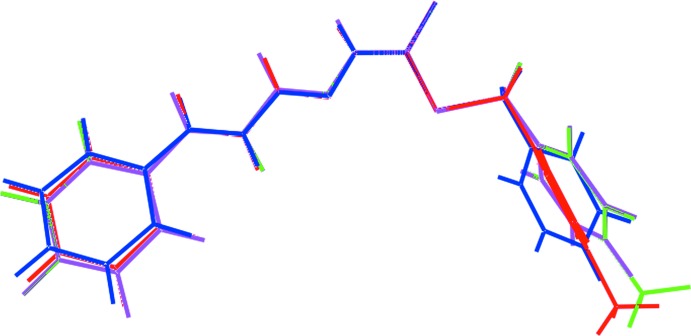
Structural overlay between the crystal and optimized structures of (I) (red image), (Io) (green), (II) blue) and (IIo) (purple).

The results, as shown from the superposition of the experimental structure and theoretical model of (I) and (II), Fig. 5 ▸, indicate that there is not much difference between the experimental and optimized structures with the r.m.s. deviation of about 0.2110 Å in the case of (I) and 0.1747 Å in the case of (II). The key geometric parameters obtained from the calculations are also listed in Table 3 ▸. The energy-minimized structures have effective mirror symmetry whereby the S-bound aryl ring is bis­ected by the plane. The bond lengths and angles for optimized-(I) and -(II) are identical indicating no influence upon the electronic structure is exerted by the addition of a methyl group in (I). Indeed, the optimized geometries for (I) and (II) are superimposable, Fig. 5 ▸. Despite the close similarity between the optimized structures, some differences are noted between the experimental and optimized structures. For example, the C1—S2 and C11—S2 bond lengths have elongated by *ca* 0.02 and 0.03 Å, respectively. In the chain, the C1—N1 bond lengths have lengthened by *ca* 0.03 Å, a difference accompanied by a contraction in the N1—N2 bond length by about the same amount. Minor differences are also noted in bond angles with widening of S1—C1—S2 and the angles subtended at the nitro­gen atoms by 2–3° with similar contractions in the C1—S1—C11 and S1—C1—N1 angles.

Apart from geometry optimization, both (I) and (II) were also subjected to computational modelling for calculation of their inter­action energies. Briefly, the crystallographic coordinates of the experimental dimeric structures of (I) and (II) connected through N—H⋯S inter­actions were used as the input without further optimization. In order to preserve the integrity of the structure for best possible estimation of the inter­action energy from the experimental model, the positions of all hydrogen atoms obtained during crystal refinement were kept unchanged, despite that this method (riding-model approximation) is commonly known to induce deviations by as much as 0.1 to 0.2 Å shorter C—H bond lengths. The respective input structures were submitted to single point inter­action energy calculation by long-range corrected ωB97XD functional combining the D2 version of Grimme’s dispersion model and the 6-31G(*d*,*p*) basis set. It has been demonstrated that the long-range corrected hybrid method can greatly reduce self-inter­action errors (Chai & Head-Gordon, 2008 ▸) and gives a better accuracy in binding energy as compared to coupled cluster calculations (Andersen *et al.*, 2014 ▸). The computed inter­action energy (*i.e*. the energy difference between the dimer and the sum of energies for the corresponding monomers) was obtained upon the correction of basis set superposition error (BSSE) by counterpoise correction. All calculations were performed in gas phase using *Gaussian09* software (Frisch *et al.*, 2016 ▸).

The dimeric species of (I) and (II) possesses the inter­action energy (*E* ^BSSE^_int_) of −12.92 and −13.86 kcal mol^−1^, respectively. The range is approximately 3.89 to 5.23 kcal mol^−1^ less than the energy computed for a pair of thio­urea dimers at the RIMP2/cc-pVDZ and cc-pVTZ levels of theory (AlDamen & Sinnokrot, 2014 ▸). Apparently, the corresponding *E* ^BSSE^_int_ energies were overestimated due to the use of the split-valence double basis set as an necessary compromise between accuracy and computational cost since the calculations involve a rather large mol­ecular system with over 80 atoms. Despite the difference, the dimer of (II) is lower in energy (*ca* 0.94 kcal mol^−1^) *cf*. (I), indicating that the former is connected by relatively stronger N—H⋯S inter­actions and hence, the dimeric aggregate in (II) is more stable. The theoretical result is in accord with the experimental data, in which the H⋯S [2.53 (2) Å] and N⋯S [3.3714 (19) Å] bond lengths are shorter and the N—H⋯S [165 (2)°] bond angle is wider in (II) *cf*. (I), Table 1 ▸.

## Synthesis and crystallization   

The following procedure was adapted from the literature (Ravoof *et al.*, 2010 ▸): *S*-4-methyl­benzyl­dithio­carbazate (2.12 g, 0.01 mol) was dissolved in hot aceto­nitrile (100 ml) and added to an equimolar amount of cinnamaldehyde (Merck, 1.32 g) in absolute ethanol (20 ml). The mixture was heated for about 2 h and was then allowed to stand overnight. The pale-brown crystals that formed were filtered and washed with absolute ethanol at room temperature. Yield: 70%. M.p. 463–466 K. Analysis: Calculated for C_18_H_18_N_2_S_2_: C, 66.22; H, 5.56; N, 8.58. Found: C, 65.87; H, 5.77; N, 9.00%. FT–IR (ATR, cm^−1^): 3102, ν(N—H); 1613, ν(C=N); 1021, ν(N—N); 749, ν(CSS).

## Refinement   

Crystal data, data collection and structure refinement details are summarized in Table 4 ▸. The carbon-bound H atoms were placed in calculated positions (C—H = 0.95–0.99 Å) and were included in the refinement in the riding-model approximation, with *U*_iso_(H) set to 1.2–1.5*U*_eq_(C). The nitro­gen-bound H atom was located in a difference-Fourier map but was refined with a distance restraint of N—H = 0.88±0.01 Å, and with *U*_iso_(H) set to 1.2*U*_eq_(N).

## Supplementary Material

Crystal structure: contains datablock(s) I, global. DOI: 10.1107/S2056989017003991/hb7666sup1.cif

Structure factors: contains datablock(s) I. DOI: 10.1107/S2056989017003991/hb7666Isup2.hkl

CCDC reference: 1537500

Additional supporting information:  crystallographic information; 3D view; checkCIF report

## Figures and Tables

**Table 1:** Hydrogen-bond geometry (Å, °) *Cg*1 and *Cg*2 are the centroids of the (C5–C10) and (C12—C17) rings, respectively.

*D*—H⋯*A*	*D*—H	H⋯*A*	*D*⋯*A*	*D*—H⋯*A*
N1—H1*N*⋯S1^i^	0.87 (2)	2.57 (2)	3.3984 (17)	158 (2)
C14—H14⋯*Cg*1^ii^	0.95	2.95	3.6749 (19)	134
C8—H8⋯*Cg*2^iii^	0.95	2.75	3.5571 (19)	143
C11—H11*B*⋯*Cg*2^iv^	0.99	2.78	3.5110 (18)	131

Symmetry codes: (i) 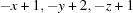; (ii) 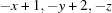; (iii) 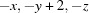; (iv) 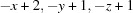.

**Table 2:** Comparison of some physical properties between (I) and (II)

Property	(I)	(II)
Volume, *V* (Å^3^)	416.41	384.29
Surface area, *A* (Å^2^)	399.66	372.94
*A*:*V*	0.96	0.97
Density, *d* (g cm^−1^)	1.274	1.320
Kitaigorodskii Packing Index, KPI (%)	67.5	68.5
Globularity, *G*	0.675	0.685
Asphericity, *Ω*	0.326	0.359

**Table 3:** Selected geometric parameters (Å, °) in (I) and (II) and in geometry-optimized-(I) and -(II)

Parameter	(I)	(II)	optimized-(I)	optimized-(II)
C1—S1	1.6752 (16)	1.670 (2)	1.665	1.665
C1—S2	1.7455 (16)	1.747 (2)	1.769	1.771
C11—S2	1.8233 (16)	1.8189 (17)	1.850	1.850
C1—N1	1.334 (2)	1.333 (2)	1.365	1.365
N1—N2	1.3845 (18)	1.382 (2)	1.354	1.353
C2—N2	1.284 (2)	1.285 (2)	1.288	1.290
C2—C3	1.435 (2)	1.433 (3)	1.439	1.439
C3—C4	1.339 (2)	1.337 (2)	1.350	1.350
				
C1—S2—C11	103.44 (7)	102.59 (9)	101.5	101.4
C1—N1—N2	120.95 (13)	120.48 (15)	122.8	122.8
N1—N2—C2	114.17 (13)	114.00 (15)	117.2	117.2
S1—C1—S2	125.20 (10)	124.67 (11)	127.0	127.0
S1—C1—N1	121.06 (12)	121.57 (13)	119.8	119.9
S2—C1—N1	113.74 (11)	113.77 (14)	113.2	113.1
C2—C3—C4	121.28 (15)	121.03 (16)	122.6	122.6
C3—C4—C5	127.33 (16)	128.25 (16)	127.5	127.5
				
S2—C11—C12—C13	106.09 (15)	−102.67 (18)	91.2	89.7
S2—C11—C12—C17	−71.41 (17)	74.56 (19)	−88.8	−90.3
C3—C4—C5—C6	−0.2 (3)	−7.0 (3)	−2.0	1.3
C3—C4—C5—C10	178.69 (16)	173.64 (19)	178.0	−178.8

**Table 4:** Experimental details

Crystal data	
Chemical formula	C_18_H_18_N_2_S_2_
*M*_r_	326.46
Crystal system, space group	Triclinic, *P* 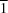
Temperature (K)	100
*a*, *b*, *c* (Å)	5.6720 (3), 12.6288 (7), 13.4690 (8)
α, β, γ (°)	62.451 (6), 84.441 (5), 88.930 (5)
*V* (Å^3^)	851.00 (9)
*Z*	2
Radiation type	Cu *K*α
μ (mm^−1^)	2.80
Crystal size (mm)	0.19 × 0.18 × 0.08

Data collection	
Diffractometer	Agilent Xcalibur, Eos, Gemini
Absorption correction	Multi-scan *CrysAlis PRO* (Agilent, 2011 ▸)
*T*_min_, *T*_max_	0.802, 1.000
No. of measured, independent and observed [*I* > 2σ(*I*)] reflections	11378, 3272, 2922
*R*_int_	0.025
(sin θ/λ)_max_ (Å^−1^)	0.614

Refinement	
*R*[*F* ^2^ > 2σ(*F* ^2^)], *wR*(*F* ^2^), *S*	0.036, 0.098, 1.03
No. of reflections	3272
No. of parameters	203
No. of restraints	1
H-atom treatment	H atoms treated by a mixture of independent and constrained refinement
Δρ_max_, Δρ_min_ (e Å^−3^)	0.38, −0.21

Computer programs: CrysAlis (Agilent, 2011 ▸), *SHELXS97* (Sheldrick, 2008 ▸), *SHELXL2014/7* (Sheldrick, 2015 ▸), *ORTEP-3 for Windows* (Farrugia, 2012 ▸), *DIAMOND* (Brandenburg, 2006 ▸) and *publCIF* (Westrip, 2010 ▸).

## supplementary crystallographic information

### Crystal data

**Table:**

C_18_H_18_N_2_S_2_	*Z* = 2
*M**_r_* = 326.46	*F*(000) = 344
Triclinic, *P*1	*D*_x_ = 1.274 Mg m^−^^3^
*a* = 5.6720 (3) Å	Cu *K*α radiation, λ = 1.5418 Å
*b* = 12.6288 (7) Å	Cell parameters from 5602 reflections
*c* = 13.4690 (8) Å	θ = 3.7–71.2°
α = 62.451 (6)°	µ = 2.80 mm^−^^1^
β = 84.441 (5)°	*T* = 100 K
γ = 88.930 (5)°	Prism, light-brown
*V* = 851.00 (9) Å^3^	0.19 × 0.18 × 0.08 mm

### Data collection

**Table:**

Agilent Xcalibur, Eos, Gemini diffractometer	3272 independent reflections
Radiation source: Enhance (Cu) X-ray Source	2922 reflections with *I* > 2σ(*I*)
Graphite monochromator	*R*_int_ = 0.025
Detector resolution: 16.1952 pixels mm^-1^	θ_max_ = 71.3°, θ_min_ = 3.7°
ω scans	*h* = −6→6
Absorption correction: multi-scan CrysAlisPro (Agilent, 2011)	*k* = −15→15
*T*_min_ = 0.802, *T*_max_ = 1.000	*l* = −16→16
11378 measured reflections	

### Refinement

**Table:**

Refinement on *F*^2^	1 restraint
Least-squares matrix: full	H atoms treated by a mixture of independent and constrained refinement
*R*[*F*^2^ > 2σ(*F*^2^)] = 0.036	*w* = 1/[σ^2^(*F*_o_^2^) + (0.063*P*)^2^ + 0.2179*P*] where *P* = (*F*_o_^2^ + 2*F*_c_^2^)/3
*wR*(*F*^2^) = 0.098	(Δ/σ)_max_ < 0.001
*S* = 1.03	Δρ_max_ = 0.38 e Å^−^^3^
3272 reflections	Δρ_min_ = −0.21 e Å^−^^3^
203 parameters	

### Special details

**Table:**

Geometry. All esds (except the esd in the dihedral angle between two l.s. planes) are estimated using the full covariance matrix. The cell esds are taken into account individually in the estimation of esds in distances, angles and torsion angles; correlations between esds in cell parameters are only used when they are defined by crystal symmetry. An approximate (isotropic) treatment of cell esds is used for estimating esds involving l.s. planes.

### Fractional atomic coordinates and isotropic or equivalent isotropic displacement parameters (Å^2^)

**Table:**

	*x*	*y*	*z*	*U*_iso_*/*U*_eq_	
S1	0.70595 (7)	0.83091 (3)	0.55216 (3)	0.02412 (13)	
S2	0.61163 (7)	0.76634 (3)	0.36831 (3)	0.02024 (13)	
N1	0.3820 (2)	0.92216 (12)	0.41092 (11)	0.0213 (3)	
H1N	0.356 (3)	0.9703 (15)	0.4406 (15)	0.026*	
N2	0.2657 (2)	0.93744 (12)	0.31965 (11)	0.0218 (3)	
C1	0.5581 (3)	0.84581 (13)	0.44483 (13)	0.0194 (3)	
C2	0.0948 (3)	1.01051 (14)	0.29902 (13)	0.0211 (3)	
H2	0.0558	1.0455	0.3474	0.025*	
C3	−0.0385 (3)	1.04062 (14)	0.20473 (14)	0.0225 (3)	
H3	−0.0020	1.0045	0.1572	0.027*	
C4	−0.2135 (3)	1.11863 (14)	0.18249 (14)	0.0226 (3)	
H4	−0.2465	1.1506	0.2336	0.027*	
C5	−0.3594 (3)	1.16011 (14)	0.08835 (13)	0.0213 (3)	
C6	−0.3306 (3)	1.12110 (15)	0.00607 (14)	0.0257 (4)	
H6	−0.2076	1.0681	0.0085	0.031*	
C7	−0.4800 (3)	1.15911 (15)	−0.07886 (14)	0.0286 (4)	
H7	−0.4600	1.1311	−0.1335	0.034*	
C8	−0.6589 (3)	1.23796 (16)	−0.08460 (14)	0.0287 (4)	
H8	−0.7615	1.2635	−0.1427	0.034*	
C9	−0.6863 (3)	1.27894 (16)	−0.00499 (15)	0.0294 (4)	
H9	−0.8071	1.3335	−0.0090	0.035*	
C10	−0.5382 (3)	1.24059 (16)	0.08037 (14)	0.0262 (4)	
H10	−0.5584	1.2695	0.1344	0.031*	
C11	0.8493 (3)	0.66793 (14)	0.43635 (13)	0.0209 (3)	
H11A	0.9914	0.7149	0.4318	0.025*	
H11B	0.7981	0.6139	0.5165	0.025*	
C12	0.9009 (3)	0.59760 (13)	0.37170 (13)	0.0186 (3)	
C13	1.1047 (3)	0.62246 (15)	0.29770 (14)	0.0233 (3)	
H13	1.2160	0.6817	0.2901	0.028*	
C14	1.1471 (3)	0.56131 (16)	0.23458 (14)	0.0260 (4)	
H14	1.2876	0.5793	0.1845	0.031*	
C15	0.9884 (3)	0.47474 (14)	0.24348 (13)	0.0226 (3)	
C16	0.7849 (3)	0.44935 (15)	0.31824 (15)	0.0274 (4)	
H16	0.6740	0.3898	0.3261	0.033*	
C17	0.7425 (3)	0.50994 (15)	0.38120 (15)	0.0267 (4)	
H17	0.6026	0.4913	0.4318	0.032*	
C18	1.0331 (4)	0.40915 (17)	0.17430 (16)	0.0326 (4)	
H18A	1.1663	0.4481	0.1168	0.049*	
H18B	0.8910	0.4107	0.1377	0.049*	
H18C	1.0709	0.3261	0.2234	0.049*	

### Atomic displacement parameters (Å^2^)

**Table:**

	*U*^11^	*U*^22^	*U*^33^	*U*^12^	*U*^13^	*U*^23^
S1	0.0306 (2)	0.0249 (2)	0.0242 (2)	0.00799 (17)	−0.01188 (17)	−0.01611 (17)
S2	0.0252 (2)	0.0196 (2)	0.0208 (2)	0.00461 (15)	−0.00790 (15)	−0.01252 (16)
N1	0.0254 (7)	0.0217 (7)	0.0226 (7)	0.0054 (6)	−0.0082 (5)	−0.0141 (6)
N2	0.0242 (7)	0.0220 (7)	0.0204 (7)	0.0018 (6)	−0.0062 (5)	−0.0101 (5)
C1	0.0226 (8)	0.0183 (7)	0.0183 (7)	−0.0005 (6)	−0.0024 (6)	−0.0094 (6)
C2	0.0219 (8)	0.0205 (8)	0.0234 (8)	0.0005 (6)	−0.0030 (6)	−0.0121 (6)
C3	0.0247 (9)	0.0209 (8)	0.0220 (8)	0.0002 (6)	−0.0033 (6)	−0.0098 (6)
C4	0.0237 (8)	0.0230 (8)	0.0222 (8)	−0.0018 (6)	−0.0022 (6)	−0.0113 (6)
C5	0.0206 (8)	0.0190 (7)	0.0210 (8)	−0.0014 (6)	−0.0024 (6)	−0.0062 (6)
C6	0.0290 (9)	0.0219 (8)	0.0264 (9)	0.0035 (7)	−0.0059 (7)	−0.0108 (7)
C7	0.0383 (10)	0.0246 (8)	0.0227 (8)	−0.0004 (7)	−0.0067 (7)	−0.0099 (7)
C8	0.0258 (9)	0.0293 (9)	0.0228 (8)	−0.0015 (7)	−0.0078 (7)	−0.0041 (7)
C9	0.0232 (9)	0.0322 (9)	0.0271 (9)	0.0067 (7)	−0.0034 (7)	−0.0089 (7)
C10	0.0249 (9)	0.0298 (9)	0.0228 (8)	0.0037 (7)	−0.0011 (7)	−0.0117 (7)
C11	0.0228 (8)	0.0208 (8)	0.0222 (8)	0.0042 (6)	−0.0070 (6)	−0.0119 (6)
C12	0.0213 (8)	0.0172 (7)	0.0178 (7)	0.0048 (6)	−0.0057 (6)	−0.0080 (6)
C13	0.0199 (8)	0.0263 (8)	0.0256 (8)	−0.0009 (6)	−0.0040 (6)	−0.0133 (7)
C14	0.0208 (8)	0.0337 (9)	0.0247 (8)	0.0026 (7)	0.0001 (6)	−0.0150 (7)
C15	0.0283 (9)	0.0227 (8)	0.0191 (7)	0.0073 (7)	−0.0058 (6)	−0.0112 (6)
C16	0.0317 (9)	0.0233 (8)	0.0297 (9)	−0.0050 (7)	0.0008 (7)	−0.0151 (7)
C17	0.0276 (9)	0.0279 (9)	0.0275 (9)	−0.0050 (7)	0.0064 (7)	−0.0168 (7)
C18	0.0408 (11)	0.0339 (10)	0.0314 (9)	0.0077 (8)	−0.0038 (8)	−0.0222 (8)

### Geometric parameters (Å, º)

**Table:**

S1—C1	1.6752 (16)	C9—C10	1.385 (2)
S2—C1	1.7455 (16)	C9—H9	0.9500
S2—C11	1.8233 (16)	C10—H10	0.9500
N1—C1	1.334 (2)	C11—C12	1.513 (2)
N1—N2	1.3845 (18)	C11—H11A	0.9900
N1—H1N	0.873 (9)	C11—H11B	0.9900
N2—C2	1.284 (2)	C12—C17	1.390 (2)
C2—C3	1.435 (2)	C12—C13	1.389 (2)
C2—H2	0.9500	C13—C14	1.392 (2)
C3—C4	1.339 (2)	C13—H13	0.9500
C3—H3	0.9500	C14—C15	1.383 (2)
C4—C5	1.463 (2)	C14—H14	0.9500
C4—H4	0.9500	C15—C16	1.393 (2)
C5—C10	1.398 (2)	C15—C18	1.510 (2)
C5—C6	1.402 (2)	C16—C17	1.384 (2)
C6—C7	1.386 (2)	C16—H16	0.9500
C6—H6	0.9500	C17—H17	0.9500
C7—C8	1.390 (3)	C18—H18A	0.9800
C7—H7	0.9500	C18—H18B	0.9800
C8—C9	1.386 (3)	C18—H18C	0.9800
C8—H8	0.9500		
			
C1—S2—C11	103.44 (7)	C9—C10—H10	119.5
C1—N1—N2	120.95 (13)	C5—C10—H10	119.5
C1—N1—H1N	118.5 (13)	C12—C11—S2	104.86 (10)
N2—N1—H1N	119.9 (13)	C12—C11—H11A	110.8
C2—N2—N1	114.17 (13)	S2—C11—H11A	110.8
N1—C1—S1	121.06 (12)	C12—C11—H11B	110.8
N1—C1—S2	113.74 (11)	S2—C11—H11B	110.8
S1—C1—S2	125.20 (10)	H11A—C11—H11B	108.9
N2—C2—C3	121.60 (15)	C17—C12—C13	118.22 (15)
N2—C2—H2	119.2	C17—C12—C11	121.22 (14)
C3—C2—H2	119.2	C13—C12—C11	120.52 (14)
C4—C3—C2	121.28 (15)	C12—C13—C14	120.52 (15)
C4—C3—H3	119.4	C12—C13—H13	119.7
C2—C3—H3	119.4	C14—C13—H13	119.7
C3—C4—C5	127.33 (16)	C15—C14—C13	121.22 (15)
C3—C4—H4	116.3	C15—C14—H14	119.4
C5—C4—H4	116.3	C13—C14—H14	119.4
C10—C5—C6	118.13 (15)	C14—C15—C16	118.22 (15)
C10—C5—C4	119.07 (15)	C14—C15—C18	121.27 (16)
C6—C5—C4	122.79 (15)	C16—C15—C18	120.51 (15)
C7—C6—C5	120.63 (16)	C17—C16—C15	120.68 (16)
C7—C6—H6	119.7	C17—C16—H16	119.7
C5—C6—H6	119.7	C15—C16—H16	119.7
C6—C7—C8	120.42 (17)	C16—C17—C12	121.16 (15)
C6—C7—H7	119.8	C16—C17—H17	119.4
C8—C7—H7	119.8	C12—C17—H17	119.4
C9—C8—C7	119.50 (16)	C15—C18—H18A	109.5
C9—C8—H8	120.3	C15—C18—H18B	109.5
C7—C8—H8	120.3	H18A—C18—H18B	109.5
C8—C9—C10	120.21 (17)	C15—C18—H18C	109.5
C8—C9—H9	119.9	H18A—C18—H18C	109.5
C10—C9—H9	119.9	H18B—C18—H18C	109.5
C9—C10—C5	121.09 (17)		
			
C1—N1—N2—C2	177.67 (14)	C6—C5—C10—C9	1.3 (2)
N2—N1—C1—S1	177.67 (11)	C4—C5—C10—C9	−177.62 (15)
N2—N1—C1—S2	−2.77 (19)	C1—S2—C11—C12	−179.86 (10)
C11—S2—C1—N1	−178.08 (11)	S2—C11—C12—C17	−71.41 (17)
C11—S2—C1—S1	1.45 (13)	S2—C11—C12—C13	106.09 (15)
N1—N2—C2—C3	177.41 (13)	C17—C12—C13—C14	0.4 (2)
N2—C2—C3—C4	−178.70 (15)	C11—C12—C13—C14	−177.20 (15)
C2—C3—C4—C5	178.23 (15)	C12—C13—C14—C15	0.1 (3)
C3—C4—C5—C10	178.69 (16)	C13—C14—C15—C16	−0.5 (3)
C3—C4—C5—C6	−0.2 (3)	C13—C14—C15—C18	179.43 (16)
C10—C5—C6—C7	−1.7 (2)	C14—C15—C16—C17	0.4 (3)
C4—C5—C6—C7	177.21 (15)	C18—C15—C16—C17	−179.51 (17)
C5—C6—C7—C8	0.9 (3)	C15—C16—C17—C12	0.1 (3)
C6—C7—C8—C9	0.3 (3)	C13—C12—C17—C16	−0.5 (3)
C7—C8—C9—C10	−0.7 (3)	C11—C12—C17—C16	177.10 (15)
C8—C9—C10—C5	−0.2 (3)		

### Hydrogen-bond geometry (Å, º)

*Cg*1 and *Cg*2 are the centroids of the (C5–C10) and (C12—C17) rings, respectively.

**Table:**

*D*—H···*A*	*D*—H	H···*A*	*D*···*A*	*D*—H···*A*
N1—H1*N*···S1^i^	0.87 (2)	2.57 (2)	3.3984 (17)	158 (2)
C14—H14···*Cg*1^ii^	0.95	2.95	3.6749 (19)	134
C8—H8···*Cg*2^iii^	0.95	2.75	3.5571 (19)	143
C11—H11*B*···*Cg*2^iv^	0.99	2.78	3.5110 (18)	131

Symmetry codes: (i) −*x*+1, −*y*+2, −*z*+1; (ii) −*x*+1, −*y*+2, −*z*; (iii) −*x*, −*y*+2, −*z*; (iv) −*x*+2, −*y*+1, −*z*+1.
